# Effects of Mobile Health App Interventions on Sedentary Time, Physical Activity, and Fitness in Older Adults: Systematic Review and Meta-Analysis

**DOI:** 10.2196/14343

**Published:** 2019-11-28

**Authors:** Dharani Yerrakalva, Dhrupadh Yerrakalva, Samantha Hajna, Simon Griffin

**Affiliations:** 1 Primary Care Unit Department of Public Health and Primary Care University of Cambridge Cambridge United Kingdom; 2 Medical Research Council Epidemiology Unit University of Cambridge Cambridge United Kingdom; 3 Barking, Havering, and Redbridge University Hospitals Trust London United Kingdom

**Keywords:** sedentary behavior, physical activity, physical fitness, aged, mHealth, mobile apps

## Abstract

**Background:**

High sedentary time, low physical activity (PA), and low physical fitness place older adults at increased risk of chronic diseases, functional decline, and premature mortality. Mobile health (mHealth) apps, apps that run on mobile platforms, may help promote active living.

**Objective:**

We aimed to quantify the effect of mHealth app interventions on sedentary time, PA, and fitness in older adults.

**Methods:**

We systematically searched five electronic databases for trials investigating the effects of mHealth app interventions on sedentary time, PA, and fitness among community-dwelling older adults aged 55 years and older. We calculated pooled standardized mean differences (SMDs) in these outcomes between the intervention and control groups after the intervention period. We performed a Cochrane risk of bias assessment and Grading of Recommendations, Assessment, Development, and Evaluation certainty assessment.

**Results:**

Overall, six trials (486 participants, 66.7% [324/486] women; age mean 68 [SD 6] years) were included (five of these trials were included in the meta-analysis). mHealth app interventions may be associated with decreases in sedentary time (SMD=−0.49; 95% CI −1.02 to 0.03), increases in PA (506 steps/day; 95% CI −80 to 1092), and increases in fitness (SMD=0.31; 95% CI −0.09 to 0.70) in trials of 3 months or shorter and with increases in PA (753 steps/day; 95% CI −147 to 1652) in trials of 6 months or longer. Risk of bias was low for all but one study. The quality of evidence was moderate for PA and sedentary time and low for fitness.

**Conclusions:**

mHealth app interventions have the potential to promote changes in sedentary time and PA over the short term, but the results did not achieve statistical significance, possibly because studies were underpowered by small participant numbers. We highlight a need for larger trials with longer follow-up to clarify if apps deliver sustained clinically important effects.

## Introduction

### Background

Older adults spend an average 9.4 hours of their day being sedentary [1] and are not meeting current physical activity (PA) recommendations [2,3]. High sedentary time, low PA, and low fitness levels place older adults at increased risk of chronic diseases [4-10], declines in functional and cognitive health [11-13], sarcopenia [14], and premature mortality [15]. Interventions to reduce sedentary time, increase PA, and improve fitness could potentially enhance the health and well-being of older adults. However, sustained positive changes in PA [16-18] and sedentary time [19,20] beyond 12 months have not been consistently achieved through traditional interventions.

Mobile health (mHealth) apps, software apps that run on a mobile platform such as a mobile phone or tablet [21], may provide an alternative approach as they circumvent many of the limitations of traditional professional-led interventions. Common traditional interventions include advice from health care professionals and educational materials. The limitations of these methods are restricted access to professionals and limited reach. Furthermore, there is considerable cost associated with training and employing the professionals required to deliver these interventions. The duration of monitoring and feedback may also be limited by cost and time restraint, and therefore, there is no provision for frequent or any follow-up. Other traditional methods such as educational materials lack tailoring.

Apps have the potential to be more cost-effective than traditional interventions [22]; although this field is developing, cost-effectiveness analyses are not widely available. They can overcome barriers to accessing health care as they can be used independent of a health care provider, and they appeal to healthy individuals and those with medical conditions and therefore have the potential to reach a larger percentage of the population. In addition, app technologies allow GPS monitoring, tailored feedback, and reminders throughout the day. In 2017, there were 325,000 health apps available in major app stores [23]. Older adults are traditionally not seen as app users, but mobile phone use among older adults is significant and increasing [24-26], as is the use of health apps.

mHealth is defined as health interventions involving mobile devices [1], and apps are one such intervention. mHealth interventions themselves are a subset of electronic health (eHealth; an umbrella term that includes any information and communication technologies utilized in the health care field). mHealth apps confer unique advantages over other eHealth (static computer, internet, landline use, text messaging, and mobile telephone calls) as they can allow continuous monitoring that provides the basis for individualized feedback and goal setting.

Health-related apps are currently recommended in a range of health contexts. This includes the management of a variety of health conditions such as depression, dementia, diabetes, and chronic obstructive pulmonary disease [27]; medication adherence and rehabilitation; use as symptoms checkers; and managing clinical records. Apps can provide quick advice, support from peers, and the ability to self-monitor. There is a growing interest in using apps to modify behaviors such as PA or sedentariness to improve or maintain health. These apps commonly feature self-monitoring of behavior, either through self-inputting data or by linking to a monitoring device (eg, pedometer or smart watches). With these data, the apps can provide feedback, prompts, goal setting, rewards, and social connectivity.

Two recent systematic reviews investigated the effectiveness of eHealth interventions on PA [28,29], although no meta-analyses were done. Both reviews reported that eHealth interventions promote PA in the short-term among adults older than 55 years and that evidence regarding the long-term effects is still lacking.

### Objectives

There is no existing review examining the specific role of mHealth app interventions on PA in older adults. Furthermore, there is no existing systematic review or meta-analysis examining the effect of mHealth app interventions on sedentary time or fitness in older adults.

Such a review is needed to inform the development of scalable and effective activity interventions among older adults. To fill this gap in knowledge, we aimed to synthesize the existing evidence on the effectiveness of mHealth app interventions on sedentary time, PA, and fitness in older adults and to identify common behavioral change techniques (BCTs) utilized in effective interventions.

## Methods

### Protocol and Eligibility Criteria

We published a Prospective Register of Systematic Reviews (PROSPERO) protocol before undertaking this review (CRD42018106195). We included trials (randomized or nonrandomized) that compared the effectiveness of an mHealth app intervention with either a modified dose of intervention (modified volume of intervention or modified version of same app), different app, nonapp intervention, or no intervention. To be eligible, the trials had to include community-dwelling adults aged 55 years and older. The outcomes assessed in this study were PA (moderate- to vigorous-intensity physical activity [MVPA] measured by accelerometer and steps/day measured by pedometer), physical fitness (maximal oxygen uptake [VO_2_ max], gait speed, and 6-min walk), or sedentary time (sedentary time measured by accelerometer). The outcome measures had to be objectively assessed. We only included trials in the meta-analysis that reported outcome values pre- and postintervention. We limited the searches to human studies published after 2008 as the emergence of mHealth apps occurred after this time. We excluded trials that included nonapp interventions only (ie, other types of eHealth or mHealth interventions only and no apps).

### Information Sources and Search

We systematically searched 5 medical electronic databases for trials investigating effects of mHealth app interventions on sedentary time, PA, and fitness among community-dwelling older adults aged 55 years and older. These included MEDLINE via OVID, PsycINFO via Ovid, Web of Science, Cochrane Central Register of Controlled Trials, and Physical Education Index. We identified key search terms and further expanded this list by running medical subject headings searches. Search terms were classified under 3 main headings: age (aged, elderly, old, and senior), intervention (application, M-health, “mobile health”, and e-health), and outcome (sedentary, sitting, physical activity, steps, VO_2_ max, exercise, fitness, and functional aerobic capacity). We then conducted Boolean searches to systematically tie the clustered terms (and their variations through truncation) to identify potential articles (example strategy for MEDLINE in Multimedia Appendix 1). We searched for additional papers in the reference lists of review articles, protocols, key commentary articles, and the final included papers.

### Study Selection

Two authors (DAY and DRY) independently carried out all steps of the study selection and data collection detailed below. This included screening the titles and abstracts of the search results to identify articles that met the inclusion criteria and retrieving the full text of identified articles. The full-text articles were then screened. We used the Preferred Reporting Items for Systematic Reviews and Meta-Analyses guidelines (flow diagram and checklist) to ensure that we adhered to a high standard of reporting [30].

### Data Collection

We extracted data using the Cochrane Public Health Group Data Extraction and Assessment Template [31]. This included study information, participant characteristics, intervention information (duration, intensity, setting, and BCTs employed), outcomes, and risk of bias assessment information.

The BCT taxonomy [32] is used to classify BCTs employed in interventions. It was created as an *agreed language* of 93 distinct BCTs that could be used to describe the *active ingredients* in interventions. Two authors (DAY and DRY) independently underwent the Web-based training to be able to identify BCTs within interventions and then utilized this training to identify BCTs in the included studies.

### Data Analysis

For each included study, we calculated the mean difference between the postintervention outcome values of the intervention arm and the control arm. To account for the heterogeneity in the units of the outcome measures, we calculated the standardized mean difference (SMD) between the postintervention values of the intervention arm and the control. We utilized a random-effects model to estimate the pooled effect of interventions on PA, fitness, and sedentary time. Where a study reported more than one outcome, we included the measure that was most homogenous to the other included studies. We used STATA 15.0 for all analyses (StataCorp LP).

To assess heterogeneity, we calculated I² values and visually inspected forest plots. We did not examine funnel plots to examine for publication bias given that SMDs are naturally correlated with their standard errors and can produce spurious asymmetry. Furthermore, when there are less than 10 studies included in the meta-analysis, the power of the tests is too low to distinguish chance from real asymmetry.

### Risk of Bias Assessment

We assessed the internal validity of each study using the Cochrane Collaboration’s tool for assessing risk of bias [33]. Studies were categorized as having high, low, or undetermined risk of bias.

### Certainty Assessment

The results from the meta-analysis and risk of bias assessment were used to complete a Grading of Recommendations, Assessment, Development, and Evaluation (GRADE) certainty assessment [34].

## Results

### Study Selection

**Figure 1:**
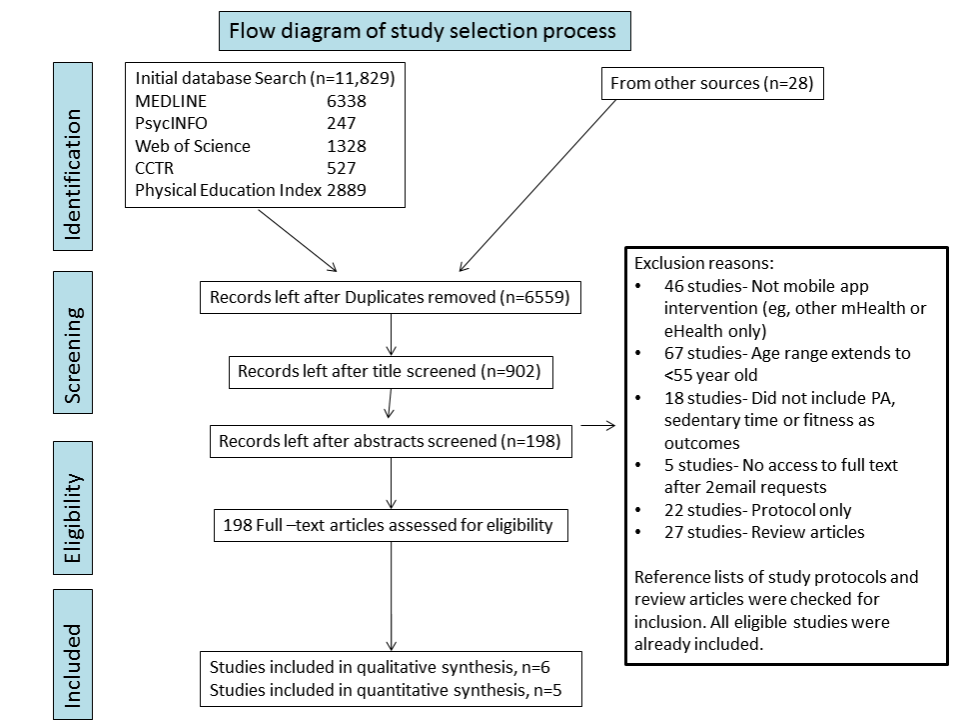
Preferred Reporting Items for Systematic Reviews and Meta-Analyses flow diagram. eHealth: electronic health; mHealth: mobile health; PA: physical activity; CCTR: Cochrane Controlled Trial Register.

There were no discrepancies between the articles retrieved or extracted data by the 2 authors. A total of 11,829 study titles were identified, with 6559 left once duplicates were removed (Figure 1). We assessed 198 full-text articles for eligibility against the inclusion criteria, with reasons listed in Figure 1. Overall, 6 studies were eligible for inclusion in the systematic review, 5 of which were eligible for inclusion in the meta-analysis.

### Study Characteristics

The included articles were published between January 2013 and March 2017. Across the 6 studies, the number of participants in each study ranged from 19 to 263 (mean 81, total N=486; Table 1). In addition, 5 studies used a parallel group randomized controlled trial (RCT) design [35-38], and 1 study was nonrandomized [39]. Studies were conducted in the United States [36,37], Switzerland [39], and Canada [35,38,40]. Participants were aged on average 68 years (SD 6), and 66.7% (324/486) of them were female. The duration of the interventions ranged from 2 to 6 months and the duration of follow-up ranged from 2 to 12 months (Table 1). None of the studies specified whether the apps, or other elements of interventions such as fitness trackers, were taken away from participants at the end of the monitored intervention or left with them to be used until follow-up.

**Table 1:** Characteristics of included studies.

First author, year	Total number of participants	Number in intervention group	Number in comparator group	Age (years), mean (SD)	Sex female, n (%)	Country	Study design	Duration of intervention (months)	Follow-up measurement (months)
Ashe, 2015 [35]	19	12	7	64 (4.6)	19 (100)	Canada	2-arm RCT^a^	6	3 and 6
Bickmore, 2013 [36]	263	132	131	71.3 (5.4)	161 (61.2)	United States	2-arm RCT	2	2 and 12
Silveira, 2013 [39]	44	Ind^b^ 14; Soc^c^ 13	17	Ind 74 (5); Soc 75 (6); Cont^d^ 76 (15)	Ind 10 (71); Soc 8 (62); Cont 10 (59)	Switzerland	2-arm non-RCT	3	3
Knight, 2014 [40]	60	SB^e^ 14; EX^f^ 15; CC^g^ 16	15	63 (4)	39 (65)	Canada	3-arm random, no control	3	3
Lyons, 2017 [37]	40	20	20	61.5 (5.6)	34 (85)	United States	2-arm RCT	3	3
Knight, 2014 [38]	60	SB 14; EX 15; CC 16	15	63 (4)	39 (65)	Canada	4-arm RCT	3	3

^a^RCT: randomized controlled trial.

^b^Ind: individual intervention group.

^c^Soc: social intervention group.

^d^Cont: control group.

^e^SB: sedentary behavior intervention.

^f^EX: physical activity intervention.

^g^CC: combined intervention.

All app interventions but one involved syncing the app to wearable technology (Table 2). Overall, 3 studies had users syncing or inputting data from pedometers [36,38,40] to apps, and 2 studies [35,37] had users syncing a wearable smart device (Fitbit [Fitbit Inc] and UP24 [Jawbone]) with apps. In 4 studies [35-37,39], app functionality allowed goal setting and tailored feedback or prompts related to progress to goals. In addition, 2 studies included apps that only allowed monitoring of step count, blood pressure, and blood glucose with no goal setting or prompts [38,40].

mHealth app interventions were delivered through smartphones in 3 studies [35,38,40] and tablet computers in 3 studies [36,37,39]. The app was the primary focus of the intervention in 3 studies [36,37,39], whereas the app was used in combination with educational classes and phone calls with health care professionals in 3 studies [35,38,40]. In addition, 2 studies had a *no-content* comparator group [37,40] (Table 2), 2 studies had *nontechnology* comparator groups [35,39], and 1 study had a *technology nonapp* comparator group [36]. In 1 study [38], all groups included mHealth app interventions, and therefore, this study was not included in the meta-analysis. Of the 6 studies, 4 reported the effectiveness of their intervention on PA [35-38], 2 on sedentary time [35,37], and 3 on physical fitness [37,39,40].

**Table 2:** Intervention and comparator group characteristics.

First author, year	Outcome (measurement)	Intervention description	App characteristics	Comparator description	Intervention frequency
Ashe, 2015 [35]	PA^a^ (minutes/day, Actigraph), sedentary time (% sedentary time/day, Actigraph)	Group education, individualized PA prescription, Fitbit with Fitbit app use	App syncs with wearable device. Can view daily steps, calories burned, active minutes, and sleep and track food and weight. Allows monitoring trends, goal setting and tailored daily prompts. Social networking forums	Educational sessions without PA component	4 weekly sessions, then 5 monthly sessions
Bickmore, 2013 [36]	PA (steps/day, pedometer)	Tablet with ECA^b^ app and pedometer	App syncs with pedometer. Can view step counts. Has *virtual coach*. Allows monitoring trends and goal setting, identifies barriers, and negotiates new goals. Gives exercise tip of the day.	Pedometer and self-monitoring	Instructed to have 1 conversation with ECA per day
Silveira, 2013 [39]	Fitness (m/s, fastest gait speed)	Introductory class with iPad and active lifestyle app (either *individual* or *social* version)	App has strength-balance training plans, with videos to support exercises. Sends praise/reward messages if there is progress in goals (eg, has a flower that grows if session is completed). Allows monitoring trends. Bulletin board to discuss progress with experts and friends in social version.	Physical exercise manual and paper log	Patient guided
Knight, 2014 [40]	PA (steps/day, pedometer)	Introduction and prescription of PA (exercise, sedentary, or both) then use of smartphone + app, pedometer, glucometer, blood pressure monitor	App syncs with blood pressure and blood glucose monitors. User manually inputs steps/day from pedometer. Allows monitoring trends	No comparator, all groups had apps	Patient guided
Lyons, 2017 [37]	PA (minutes/day, activPAL); steps/day, (pedometer), sedentary time (sitting time/day, activPAL), fitness (meters, 6-min timed walk)	iPad and app/UP24 Jawbone wearable device. Initial visit then telephone counseling	Syncs with wearable device. Allows monitoring trends of step count, heart rate, sleep, food, and weight. Has a *smart coach* that offers tailored advice based on progress, goal setting, and prompts.	Control, no intervention *wait list*	Weekly telephone counseling
Knight, 2014 [38]	Fitness (maximal oxygen uptake)	Introduction and prescription for a specific intensity of PA (exercise, sedentary, or both) then use of smartphone + app, pedometer, glucometer, blood pressure monitor)	App syncs with blood pressure and blood glucose monitors. User manually inputs steps/day from pedometer. Allows monitoring trends	Control, no intervention	Patient guided

^a^PA: physical activity.

^b^ECA: embodied conversational agent.

### Effectiveness of Interventions

**Figure 2:**
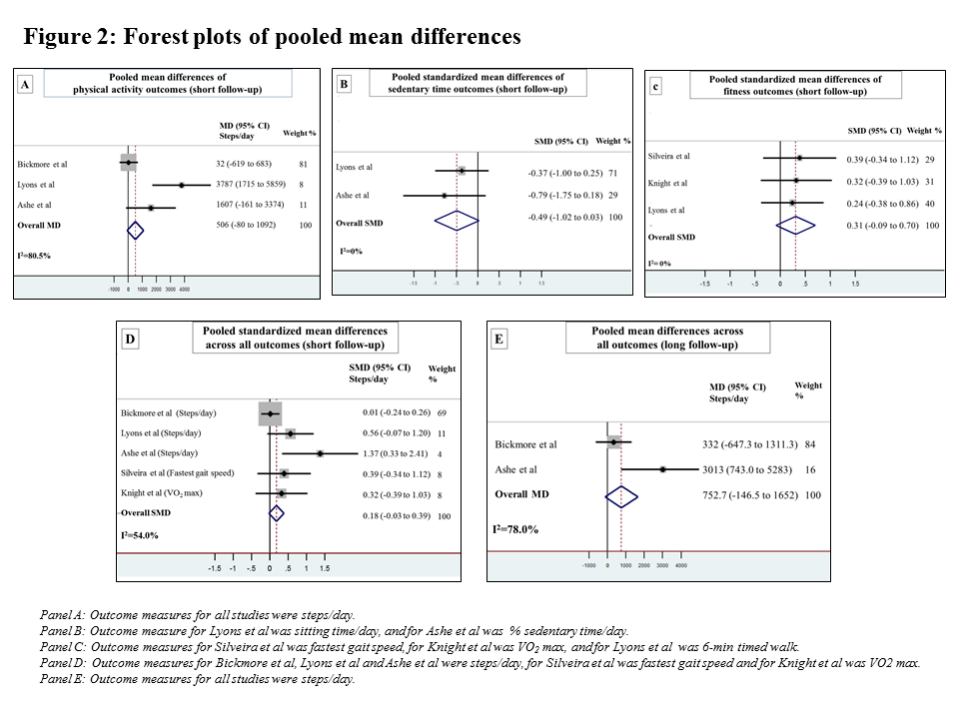
Forest plots of pooled mean differences . MD: mean difference; SMD: standardized mean difference; VO2 max: maximal oxygen uptake.

The pooled SMD across sedentary time, PA, and fitness outcomes was 0.18 (95% CI −0.03 to 0.39; Figure 2). There was evidence of statistical heterogeneity (I^2^=54%).

### Sedentary Time

#### Shorter-Term Effects (≤3 Months)

The pooled SMD for sedentary time across the 2 studies was −0.49 (95% CI −1.02 to 0.03) with no statistical heterogeneity (I^2^=0%). The effect of mHealth app interventions was inconclusive for the studies by Lyons et al [37] (−60.5 min/day in sitting time; 95% CI −161 to 40) and Ashe et al [35] (−5.1% sitting time per day; 95% CI −10.8 to 0.31).

#### Longer-Term Effects (≥6 Months)

Ashe et al [35] reported an inconclusive effect on percentage sedentary time per day over 6 months of follow-up (−1.06% sitting time/day; 95% CI −6.35 to 4.23).

### Physical Activity

#### Shorter-Term Effects (≤3 Months)

mHealth app interventions led to an average increase of 506 steps/day (pooled mean difference 95% CI −80 to 1092) across the 3 studies reporting this outcome [35-37]. Although all of the individual effect estimates were in the same direction, the effect estimates ranged greatly (from 32 to 3787 steps/day), and the pooled effect did not reach statistical significance. Statistical heterogeneity was high (I^2^=80.5%). Ashe et al [35] reported 2 measures of PA; but we only included steps/day in the pooled mean difference estimate to maximize homogeneity. Ashe et al reported a 22.1 min/day increase in MVPA (95% CI 6.64 to 37.5) [35].

The study by Knight et al [38] was not included in the pooled analysis as there was no control group. In their study, the authors examined the effect of 3 different interventions (1 intervention targeting sedentary time, 1 targeting PA, and 1 targeting both) on steps/day. The effects of all 3 interventions were inconclusive. Mean changes from baseline to 3 months were 460 steps/day (95% CI −278 to 1199) for the sedentary time intervention, −76 steps/day (95% CI −791 to 640) for the PA intervention, and −454 steps/day (95% CI −1134 to 225) for the combined intervention.

#### Longer-Term Effects (6-12 Months)

mHealth app interventions led to an average increase of 753 steps/day (pooled mean difference 95% CI −147 to 1652) across the 2 studies reporting this outcome [35,36] (Figure 2). The level of heterogeneity was high (I^2^=78%). Ashe et al [35] reported a mean increase of 3013 steps/day (95% CI 743 to 5283) and 19.6 min/day increase in MVPA at 6 months (95% CI 2.2 to 36.9). Bickmore et al [36] reported a mean increase of 332 steps/day (95% CI −647 to 1311) at 12 months.

### Physical Fitness

#### Shorter-Term Effects (≤3 Months)

The pooled SMD for fitness across the 3 studies was 0.31 (95% CI −0.09 to 0.70) with no evidence of statistical heterogeneity (I^2^=0%). The individual studies reported that their app interventions had mixed effects on fitness. Silveira et al [39] reported that their intervention led to a 0.47 m/s increase in fastest gait speed (95% CI 0.26 to 0.68). Lyons et al [37] reported a 68.3-m increase in 6-min timed walk (95% CI −106 to 243). Knight et al [40] reported mixed effects on VO_2_ max for their sedentary time intervention (−2.71 mL/min/kg; 95% CI −7.05 to 1.63), their PA intervention (+2.06 mL/min/kg; 95% CI −3.20 to 7.32), and their combined intervention (+1.98 mL/min/kg; 95% CI −2.4 to 6.36).

#### Longer-Term Effects (6-12 Months)

No studies reported on this.

### Behavioral Change Techniques

Of the 93 potential BCTs, only 31 were employed in the included studies. Studies included an average of 12 BCTs for intervention groups (range 5-21) and less than or equal to 2 BCTs in comparator groups (Multimedia Appendix 2).

The studies by Ashe et al [35] (PA), Lyons et al [37] (PA), and Silveira et al [39] (fitness, individual app) appeared to have the most effective interventions. Both the studies by Ashe et al and Lyons et al utilized apps that were linked to smart activity trackers. Furthermore, frequently employed BCTs in these effective interventions were *goal setting* (100%), *self-monitoring* (80%), *instructions on how to perform the behavior* (80%), *social reward* (80%), *social support* (60%), and *risk communication* (60%).

## Discussion

### Principal Findings

mHealth app interventions may be associated with decreases in sedentary time (SMD=−0.49), increases in PA (506 steps/day), and increases in fitness (SMD=0.31) in trials 3 months or shorter and with increases in PA (753 steps/day) in trials 6 months or longer. Results for all individual outcomes revealed trends in the same direction, but all results were inconclusive as the confidence intervals included zero.

**Figure 3:**
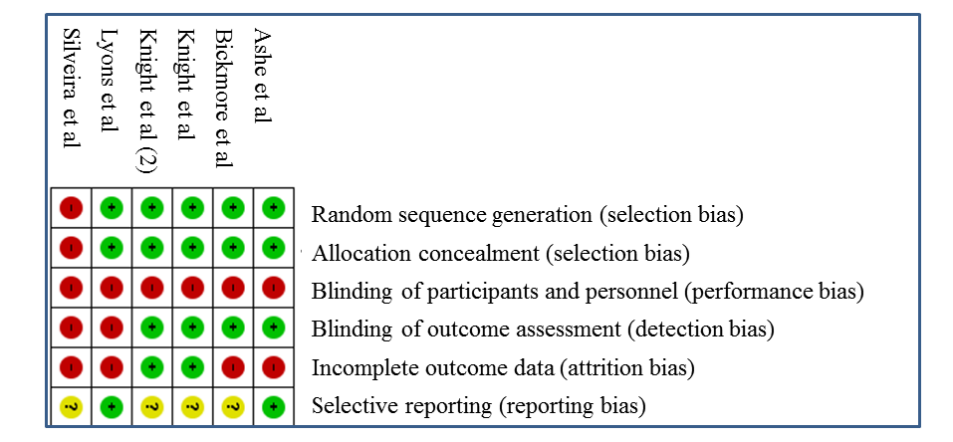
Risk of bias summary.

Overall, risk of bias was low for all studies apart from the one by Silveira et al that was a nonrandomized trial (Multimedia Appendix 3, Figure 3), the results of which may have been subject to both selection and detection bias. The majority of studies had an RCT design (5/6 studies) and described random sequence generation (5/6 studies), allocation sequence generation (5/6 studies), and blinded outcome assessment (4/6 studies). All studies were judged at high risk of performance bias as it was not possible to blind participants to an mHealth app intervention. Generally, the study attrition rates were low. In addition, 2 studies were registered in a clinical trial registry [35,37], but the remaining 4 studies did not publish a protocol or register their trial [36,38-40].

There are a number of factors that contributed to heterogeneity. These include variation in comparator groups (active and nonactive), variation in the intervention package, the length of follow-up, and variation in the outcome measurements.

For PA, the statistical heterogeneity for the pooled estimate was high (I^2^=80.5%). We tried to minimize heterogeneity by including the most homogenous measures (steps/day), but we identified a number of other sources of heterogeneity that could account for the I^2^ value. The effect estimates across the studies were varied (3787 steps/day in the study by Ashe et al [35], 1607 steps/day in the study by Lyons et al [37], and 32 steps/day in the study by Bickmore et al [36]), and the greater success of the interventions by Ashe et al and Lyons et al may have been due to a number of factors.

Although users in all 3 studies synced the app to wearable devices, apps that were synced to smart activity trackers rather than pedometers appeared to be more effective (Bickmore et al included a simple pedometer, whereas Ashe et al and Lyons et al used smart activity trackers). Furthermore, intervention packages with apps and some professional input seemed to be more effective than without (the study by Ashe et al included face-to-face group educational sessions, that by Lyons et al included weekly telephone counseling, and that by Bickmore et al had no health professional–led components). The nature of the comparator groups also appeared to influence the results. Participants in the comparator group in the study by Ashe et al had group education sessions not related to PA; in the study by Lyons et al, they underwent no intervention, whereas in the study by Bickmore et al, they were issued with pedometers. Finally, the studies by Ashe et al and Lyons et al had 3-month interventions, whereas the study by Bickmore et al had only 2-month interventions. Altogether, this may have led to Ashe et al and Lyons et al reporting larger effect sizes than Bickmore et al and hence contributed to statistical heterogeneity.

For sedentary time, there was little heterogeneity between the studies by Ashe et al and Lyons et al (I^2^=0%), with both studies including smart wearable devices in their interventions and using nonactive comparator groups.

For physical fitness, statistical heterogeneity was zero. The pooled estimate utilized 3 different measures of fitness (6-min timed walk, VO_2_ max, and fastest gait speed), although we used a standardized estimate to minimize the effect of this.

The overall GRADE certainty assessment of evidence for PA and sedentary time was moderate (Multimedia Appendix 4) and for physical fitness was low. The low certainty estimate for fitness was because the design of 1 of the 3 included trials was nonrandomized, the risk of bias for this same trial was high and risk of imprecision because of small sample size was high across all 3 studies.

Only 2 studies across this review had findings that reached statistical significance [35,39]. Common BCTs to both studies included goals and planning, feedback and monitoring, social support, and reward and threat. Both of these studies utilized wearable technologies that synced to the apps. Interestingly though, there are a number of emerging mHealth apps that use GPS technology on phones to track step count without needing wearable technology, but we found no studies testing their effectiveness in older adults.

### Comparison With the Literature

A total of 3 reviews in the literature are relevant to this review. First, in their meta-analysis, Direito et al examined the effectiveness of mHealth interventions on PA and sedentary time in adults of all ages [41]. Second, in their systematic reviews, Muellman et al [28] and Jonkman et al [29] examined the effectiveness of eHealth interventions on just PA in older adults.

Direito et al [41] included 21 RCTs (n=700, objectively measured PA). Overall, 7 of these 21 interventions included mHealth apps (the rest included other mHealth), and only 1 of these studies included older adults (Knight et al [38]). They found that mHealth interventions led to decreased sedentary time compared with control (SMD=−0.26; 95% CI −0.53 to 0). Although in the same direction and a similar effect size to our result, their result was statistically significant. Direito et al [41] examined BCTs utilized across the interventions and found that a smaller range of BCTs was employed in interventions (mean 6.9, range 2-12) and a larger range in the control group (mean 3.1, range 0-10) in comparison with our findings. This may be because app technology (in comparison with all other mHealth technology) has the potential to offer more BCTs with minimal increase in time/cost. Only 33% of the included studies by Direito et al utilized apps compared with all of our included studies.

In their narrative systematic reviews, Muellman et al [28] and Jonkman et al [29] concluded that eHealth interventions (including internet-based, telephone-based, and text messaging–based interventions) can promote PA in the short term among adults older than 55 years, but evidence on long-term effects is still lacking. Our meta-analysis was inconclusive in supporting this finding, but we observed a trend of mHealth app interventions leading to increases in step count. Only 1 of the 4 studies [36] that we included in our review reporting PA outcomes was included in the reviews by Muellman et al and Jonkman et al. What could the trends we report here mean in the context of the average behavior levels of an older adult? In their review, Tudor-Locke et al [42] found that healthy older adults take an average of 2000 to 9000 steps/day, a very broad range reflecting the natural diversity of abilities common to older adults. In addition, they concluded that 30 min of daily MVPA accumulated in addition to habitual daily activities in healthy older adults is equivalent to taking approximately 7000 to 10,000 steps/day. We report that mHealth app interventions led to a trend of increasing step count (an extra 753 steps/day). If mHealth app interventions could provide an extra 753 steps/day over the longer term, this may represent over 10% (753/7000) of the step count, which Tudor-Locke et al equate to required daily PA levels. This could be a small but potentially clinically significant change at the population level. Berkemeyer et al [43] reported that men and women aged 60 to 70 years spent 25 min/day and 19 min/day, respectively, doing MVPA (>2020 counts per minute threshold). In this context, the mean increase of 22 min/day in MVPA following an mHealth app intervention reported by Ashe et al [35] would be enough to get individuals to meet activity guidelines. However, as only 1 study reported MVPA as an outcome and the 2 other studies reporting PA reported an inconclusive effect, more studies are needed to reach firm conclusions on whether mHealth app interventions may lead to clinically significant increases in PA levels.

### Strengths and Limitations

The current meta-analysis is the first to assess the effectiveness of mHealth app interventions on sedentary time, PA, and fitness in older adults. We report here a reproducible and strong review of the current evidence, having published a prospective protocol with PROSPERO, utilized Cochrane and GRADE protocols, and undertaken a comprehensive search. We highlight the limited the number of primary studies and sample sizes in this area of research. We sought to describe sources of heterogeneity across intervention packages, comparator groups (active and nonactive), duration of intervention, and length of follow-up in detail.

The included populations limit generalizability of these results. We set a low cutoff point for our definition of older adults, which may limit generalizability to *older* adults. Only 2 studies included adults older than 79 years. All interventions were delivered in high-income countries, with only 1 European country and no countries from Asia or Africa. The focus of this review was community-dwelling older adults. Given the nature of the intervention, all of the included studies excluded individuals with severe illnesses or disease limiting the ability to walk. Therefore, this review primarily represents a healthier older population. In addition, the studies by Ashe et al, Bickmore et al, and Lyons et al had inclusion criteria targeting particularly inactive adults (eg, <60 min PA/week or no PA usually). Inactive adults may have stronger habits to initially change but may have greater opportunity for change.

### Conclusions and Implications

Mobile app interventions may be effective in decreasing sedentary time, increasing PA, and increasing fitness in trials 3 months or shorter and increasing PA in trials 6 months or longer, but we cannot conclude changes with certainty. Features that appeared to be common to effective app interventions included syncing to smart activity monitors; employing BCTs such as goal setting, self-monitoring, instructions on how to perform the behavior, and social reward; and combining apps with professional support. We found that the effectiveness of mHealth app interventions on sedentary time, PA, and fitness has been evaluated in very few studies. Furthermore, those studies that exist have small sample sizes. This review indicates the need for larger, robust RCTs into mHealth app interventions in older adults to power a future meta-analysis to reach firm conclusions on the effectiveness of mHealth app interventions in producing sustained clinical important changes in sedentary time, PA, and fitness levels.

We need to clarify if apps are associated with behavior change in the short term and, more importantly, the degree to which the changes are sustained.

With increasingly time-pressured health care systems, mHealth app interventions that can be tailored, yet delivered fast and cheaply, are potentially useful. Although technology-based interventions are becoming more commonplace in the general adult population, there is a need for a stronger evidence base to underpin interventions and for targeting older adults. The opportunity to utilize mHealth app interventions in older adults, significant numbers of whom now carry smartphones, should not be missed. We report that mHealth app interventions are a relatively underexplored tool to change PA, sedentary time, and physical fitness in older adults and recommend more attention to their utilization.

## Appendix

Multimedia Appendix 1 Literature search strategy: MEDLINE.

Multimedia Appendix 2 Summary of BCTs.

Multimedia Appendix 3 Risk of Bias Descriptions.

Multimedia Appendix 4 GRADE Certainty Assessment.

## Abbreviations

BCT: behavioral change technique
eHealth: electronic health
GRADE: Grading of Recommendations, Assessment, Development, and Evaluation
mHealth: mobile health
MVPA: moderate- to vigorous-intensity physical activity
NIHR: National Institute for Health Research
PA: physical activity
PROSPERO: Prospective Register of Systematic Reviews
RCT: randomized controlled trial
SMD: standardized mean difference
VO2 max: maximal oxygen uptake
