# Supplementary material for: Effects of Mobile Health App Interventions on Sedentary Time, Physical Activity, and Fitness in Older Adults: Systematic Review and Meta-Analysis
Source: J Med Internet Res. 2019 Nov 28;21(11):e14343. doi: 10.2196/14343 (PMC6908977; doi:10.2196/14343)
Supplement: Multimedia Appendix 1 [file jmir_v21i11e14343_app1.docx]

**Multimedia Appendix 1: Literature search strategy: MEDLINE**

Database searched: MEDLINE

Platform: Pubmed

Search undertaken January 2019

**After initial searches with Medical Subject Headings, the following search terms were used.**

1. aged
2. elder*
3. old*
4. senior*
5. adult
6. veteran*
7. geriatric*
8. retire*
9. 1 OR 2 OR 3 OR 4 OR 5 OR 6 OR 7 OR 8
10. app OR apps OR app-based
11. Mhealth or m-health
12. Smartphone* or smart phone* or mobile phone* or cell phone* or cellphone*
13. e-health OR ehealth
14. 11 OR 12 OR 13
15. sedentar* OR sitting OR inactiv*
16. activ*
17. walk*
18. strength*
19. exercise
20. MVPA
21. step OR steps
22. fit OR fitness
23. Vo2
24. "Maximal oxygen uptake"
25. 15 OR 16 OR 17 OR 18 OR 19 OR 20 OR 21 OR 22 OR 23 OR 24
26. 9 AND 14 AND 25
27. Limit 26 to yr=“2004-current”
28. Limit 27 to human
