# Supplementary material for: Effects of Mobile Health App Interventions on Sedentary Time, Physical Activity, and Fitness in Older Adults: Systematic Review and Meta-Analysis
Source: J Med Internet Res. 2019 Nov 28;21(11):e14343. doi: 10.2196/14343 (PMC6908977; doi:10.2196/14343)
Supplement: Multimedia Appendix 2 [file jmir_v21i11e14343_app2.docx]

**Multimedia Appendix 2: Summary of BCTs**

| **Study** | **Number of Control BCTs** | **Number of Intervention BCTs** | | | | | | | | | | | | **Total Number of Intervention BCTs** |
| --- | --- | --- | --- | --- | --- | --- | --- | --- | --- | --- | --- | --- | --- | --- |
|  |  | **Goals & planning** | **Feedback & monitoring** | **Social support** | **Shaping knowledge** | **Natural consequences** | **Comparison of behaviour** | **Associations** | **Repetition & substitution** | **Comparison of outcomes** | **Reward & threat** | **Antecedents** | **Self-belief** |  |
| **Ashe 2015**^33^ | 0 | 5 | 2 | 1 | 1 | 2 | 1 | 0 | 3 | 0 | 2 | 1 | 1 | 19 |
| **Bickmore 2013**^34^ | 2 | 5 | 1 | 0 | 1 | 0 | 0 | 1 | 0 | 0 | 1 | 1 | 0 | 10 |
| **Silveira 2013**^37^ | 2 | 1 | 1 | 1 | 1 | 1 | 2 | 0 | 1 | 0 | 3 | 1 | 0 | 12 |
| **Knight 2014**^38^ | 0 | 1 | 2 | 1 | 1 | 0 | 0 | 0 | 0 | 0 | 0 | 1 | 0 | 5 |
| **Lyons 2017**^35^ | 0 | 5 | 3 | 2 | 2 | 3 | 1 | 1 | 1 | 1 | 1 | 1 | 0 | 21 |
| **Knight 2014**^36^ | 0 | 1 | 2 | 1 | 1 | 0 | 0 | 0 | 0 | 0 | 0 | 1 | 0 | 5 |

This table summarises the BCTs which were used in each study under the headings outlined in the BCT Taxonomy^30^.
