# Supplementary material for: Effects of Mobile Health App Interventions on Sedentary Time, Physical Activity, and Fitness in Older Adults: Systematic Review and Meta-Analysis
Source: J Med Internet Res. 2019 Nov 28;21(11):e14343. doi: 10.2196/14343 (PMC6908977; doi:10.2196/14343)
Supplement: Multimedia Appendix 3 [file jmir_v21i11e14343_app3.docx]

**Multimedia Appendix 3: Risk of bias descriptions**

| **Author(year)** | **ITT Analysis** | **Attrition bias/rates** | **Missing data analysis** | **Selective reporting bias (Protocol/Trial registration)** | **Random sequence generation** | **Allocation Concealment** | **Blinding of Outcome Assessment** | **Overall Risk** |
| --- | --- | --- | --- | --- | --- | --- | --- | --- |
| Ashe (2015)^33^ | No | Intervention group 92% completed, control group 67% completed | Not mentioned | ClinicalTrials.gov NCT01842061. | Yes | Yes | Yes | Low risk |
| Bickmore (2013)^34^ | Yes | 86% completed, reported “no significant difference between attrition for intervention and control groups” | Analysis for effect of missing data, no significant impact | None | Yes | Yes | Yes | Low risk |
| Silveira (2013)^37^ | No | Individual intervention group 79% completed,  social intervention group 92% completed,  control group 59% completed | Not mentioned | None | No | No | No | High risk |
| Knight (2014)^38^ | No | 98% completed, reported “no significant difference between groups” | Not mentioned | None | Yes | Yes | Yes | Low risk |
| Lyons (2017)^35^ | Yes | Intervention group 95% completed, control group 95% completed | No analysis | Clinicaltrials.gov NCT01869348 | Yes | No | No | Unclear risk |
| Knight(2014)^36^ | No | 98% completed, reported “no significant difference between groups” | Not mentioned | None | Yes | Yes | Yes | Low risk |
