# Supplementary material for: Effects of Mobile Health App Interventions on Sedentary Time, Physical Activity, and Fitness in Older Adults: Systematic Review and Meta-Analysis
Source: J Med Internet Res. 2019 Nov 28;21(11):e14343. doi: 10.2196/14343 (PMC6908977; doi:10.2196/14343)
Supplement: Multimedia Appendix 4 [file jmir_v21i11e14343_app4.docx]

| **Certainty Assessment** | | | | | | **No of patients** | | SMD | **Certainty** | | **Importance** |
| --- | --- | --- | --- | --- | --- | --- | --- | --- | --- | --- | --- |
| **No of studies** | **Design** | **Risk of bias** | **Inconsistency** | **Indirectness** | **Imprecision** | **Intervention** | **Control** |  |  |  |  |
|  |  |  |  |  |  |  |  |  |  |  |  |
| **Outcome: Fitness** | | | | | | | | | | | |
| 3 | 2 randomised trials, 1 non-randomised *(3)* | Serious- High risk of bias for 1 study, low risk of bias for 2 studies *(-0.5)* | No serious inconsistency,I² 0%) | No serious indirectness | Serious risk of imprecision as small sample size *(-0.5)* | 50 | 52 | 0.31 (95%CI -0.09, 0.70) | Low | ⊕⊕ | Important |
|  |  |  |  |  |  |  |  |  |  |  |  |
| **Outcome: Physical Activity** | | | | | | | | | | | |
| 3 | Randomised trials *(4)* | No change-overall low/unclear risk of bias for all studies | No serious inconsistency,I² =67.6% | No serious indirectness | Serious risk of imprecision as small sample size *(-0.5)* | 164 | 158 | 0.14 (95% CI -0.09, 0.36) | Moderate | ⊕⊕⊕ | Important |
| **Outcome: Sedentary Time** | | | | | | | | | | | |
| 2 | Randomised trials *(4)* | No serious risk of bias | No serious inconsistency, I² 0% | No serious indirectness | Serious risk of imprecision as small sample sizes *(-0.5)* | 32 | 27 | −0.49 (95% CI −1.02, 0.03) | Moderate | ⊕⊕⊕ | Important |

**Multimedia Appendix 4: GRADE Certainty Assessment**

GRADE scoring is in italics. Randomised-control trials provide the highest starting score (4), with deductions thereafter for high risk of bias, inconsistency, indirectness and imprecision.
